# High-Sensitivity CRP and Occurrence of Cancer in Cardiovascular Disease Patients with Cardiovascular

**DOI:** 10.3390/jcm14041193

**Published:** 2025-02-12

**Authors:** Orianne de la Brassinne Bonardeaux, Manon Deneye, Cecile Oury, Marie Moonen, Patrizio Lancellotti

**Affiliations:** 1 Department of Cardiology, GIGA Cardiovascular, University of Liège Hospital, 4000 Liège, Belgium; ori.dlbbonardeaux@student.uliege.be (O.d.l.B.B.);; 2 Cardiology Department, Hospital Center of Bois de l’Abbaye, 4100 Seraing, Belgium; 3 Laboratory of Cardiology, Department of Cardiology, GIGA Cardiovascular Sciences, University of Liège Hospital, CHU Sart Tilman, 4000 Liège, Belgium

**Keywords:** cardiovascular disease, cancer, hs-CRP, antiplatelet, statin

## Abstract

**Background:** Many studies recognize a close link between inflammation, cardiovascular disease (CVD), and oncological diseases. High-sensitivity C-reactive protein (hs-CRP), a marker of low-grade systemic inflammation, is a shared feature of these conditions. This retrospective study aims to assess the predictive value of hs-CRP for the development of cancer in patients with CVD. **Methods**: Analyzing data from 174 patients undergoing coronary angiography, we assessed hs-CRP levels and collected demographic, biological, and therapeutic data that could influence the studied parameters. **Results**: Only smoking and dyslipidemia correlated significantly with CRP levels (*p* = 0.018 and 0.049, respectively). However, hs-CRP did not predict cancer development (*p* = 0.52) but correlated with 1-year and follow-up mortality (*p* = 0.011 and 0.021, respectively). Antiplatelet and statin use was higher in the cancer-free group and associated with a lower probability of developing cancer (*p* < 0.001 and *p* = 0.009, respectively). **Conclusions**: While hs-CRP did not prove effective as a cancer predictor in our study, it correlated with all-cause mortality. Our findings suggest a potential protective effect of antiplatelet and statin treatments against cancer development, prompting further research to understand underlying processes and identify key factors in the pathophysiology of these diseases.

## 1. Introduction

Cardiovascular diseases (CVD), a leading global cause of morbidity and mortality, primarily result from atherosclerosis, a complex and incompletely understood process [1]. Inflammation has been firmly established as a key player in atherogenesis [2]. Indeed, cardiovascular risk factors initiate endothelial activation. This triggers an inflammatory cascade involving lipoproteins, immune cells, and cytokines. Plaque forms and may rupture, causing thrombosis that can manifest as myocardial infarction, peripheral ischemia, and stroke. These are the cardiovascular manifestations of atheromatous processes leading to tissue hypoxia [3,4,5].

With this in mind, growing evidence supports the use of biomarkers as indicators or predictors of atherosclerotic complications. Potential markers include oxidized low density lipoproteins (LDL), proinflammatory cytokines (especially interleukin 1, interleukin 6, and tumor necrosis factor), adhesion molecules, and hepatic synthesis products such as CRP. Additionally, the elevation of white blood cell count, particularly T cells, serves as an indicator of the cellular response to inflammation [6].

CRP is a pentameric protein in the pentraxin family [7]. It is the classic acute-phase inflammation marker, with roles in the classical complement pathway, leukocyte activation, and phagocytosis [8]. High-sensitivity CRP (hs-CRP) rapidly increases in an inflammation setting. Assays link it to cardiovascular risk, validated by large studies showing its strong, independent predictiveness for cardiovascular risk in apparently healthy individuals and recurrence prognosis [9,10,11,12]. Moreover, studies reveal direct hs-CRP production within coronary artery smooth muscle cells, implicating it in the atherogenic process [13]. Although other cytokines, selectins, and adhesion molecules could predict clinical disease, practical issues hinder their clinical use [2,6,14]. Therefore, current evidence favors hs-CRP as the preferred analytical choice.

The measurement of hs-CRP, to reduce intra-individual variability, should be conducted in metabolically stable individuals without obvious inflammatory or infectious conditions. The American Heart Association (AHA) offers recommendations for interpreting hs-CRP levels in assessing cardiovascular risk [15]: low risk: <1 mg/L, moderate risk 1–3 mg/L, high-risk: >3 mg/dL.

It is important to note that elevated hs-CRP levels may also be associated with various factors such as overweight, heavy smoking or alcohol consumption, and pathological conditions like atrial fibrillation and heart failure [16]. Currently, screening adults for hs-CRP levels is not recommended as a public health measure. The AHA suggests measuring hs-CRP in primary prevention alongside other cardiovascular risk factors to refine risk stratification, although this remains optional and is associated with a moderate level of evidence. This analysis is particularly relevant for intermediate-risk categories [17].

In secondary prevention, hs-CRP measurement has a limited impact on therapeutic strategies. It helps in assessing the risk of recurrent events, such as death, myocardial infarction, or restenosis following percutaneous coronary intervention. In patients taking statins, hs-CRP has been shown to be a stronger predictor of future cardiovascular events and mortality than LDL-C [18].

Chronic inflammation is now recognized as a potential cause of malignancy and was first described by Virchow [19]. It is linked to DNA damage through various mechanisms and associated with the progression and severity of many cancers [20]. Currently, two signaling pathways link inflammation and cancer: intrinsic and extrinsic. The intrinsic pathway is comparable to chronic inflammatory diseases such as atherosclerosis. In a persistent inflammation state, high amounts of cytokines are produced. These cytokines trigger intracellular signaling. This increases the transcription of proinflammatory cytokines. It also produces free radicals, and proteolytic enzymes. These changes alter oncogene and tumor suppressor gene expression. A self-perpetuating cycle of cellular chemotaxis and cytokine release follows [21]. It is important to note that we cannot assert that all inflammatory processes always have pathological consequences.

With this in mind, the connection between CVD, inflammation, and cancer seems well-established [22]. Multiple factors, including overweight, smoking, alcohol, and certain pathological conditions like atrial fibrillation or heart failure, can contribute to inflammation. Shared risk factors between CVD and neoplastic conditions imply that low-grade inflammation may represent a common pathway leading to both. Hs-CRP serves as a marker for low-grade systemic inflammation. It is already known to predict recurrence in CVD patients. A study from van’t Kloster suggests that it could also predict the risk of developing cancer in CVD patients [23].

Based on that study, our objective was to investigate the relationship between low-grade systemic inflammation, represented by the hs-CRP biological assay, in patients with CVD and the occurrence of an oncological condition during their follow-up.

## 2. Materials and Methods

This was a single-center cross-sectional study analyzing the demographic, medical (personal history, cardiovascular risk factors), biological (including inflammatory parameters), and therapeutic data of patients with CVD.

### 2.1. Population

We initially included 260 patients. Inclusion criteria were having undergone coronary angiography and hs-CRP measurement during their day hospitalization for this technical procedure between October 2013 and April 2017 at the university hospital of CHU Sart Tilman, Liège, Belgium. From this group of patients, all those who developed a neoplastic condition during their medical follow-up were selected. Subsequently, we recruited corresponding patients, this time without the development of neoplasia during their follow-up, using a data matching method (1 to 1). Matching criteria were specifically chosen to analyze hs-CRP levels between the oncologic and non-oncologic groups. The criteria were chosen to minimize potential confounding factors that could influence hs-CRP levels independently of cancer status (Table 1).

**Figure 1:**
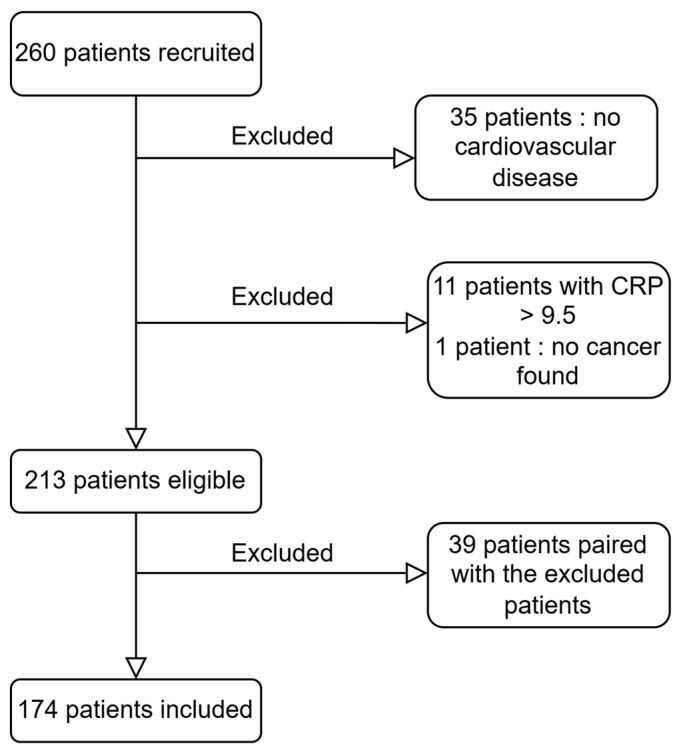
Flow chart.

Exclusion criteria were, first, the absence of a post-procedural CVD diagnosis meaning no history of stroke, peripheral vascular disease, significant coronary artery disease either before or after the coronary angiography procedure (significant coronary lesions defined as stenoses greater than 70% on coronary angiography) and second, an hs-CRP level exceeding 9.5 mg/L (revealing an ongoing inflammatory process). Patients from the non-oncological population matched to patients excluded from the oncological population were also excluded. The final number of patients studied was 174 (Figure 1).

### 2.2. Laboratory Analyses

The required blood samples were obtained routinely during each patient’s hospitalization for coronary angiography through venipuncture. The hs-CRP immunodetection was performed using the turbidimetric or immunoturbidimetric methods with Abbott reagent kits on ALINITY C (Abbott Laboratories, IL, USA). The use of quantitative liquid-phase immunoprecipitation methods (immunonephelometry and immunoturbidimetry) is recommended due to their rapid execution and automation capabilities. Laboratories typically prefer the immunoturbidimetric technique due to its low cost and suitability for use on a standard analyzer [7].

Other parameters reflecting inflammation were also recorded, such as the white blood cell count, the differential, and fibrinogen. Additional biological assays included a complete blood count, urea, creatinine, glomerular filtration rate (calculated according to the MDRD formula), cardiac markers, and lipid profile.

### 2.3. Statistical Analyses

Descriptive statistics are presented as follows: Categorical variables are shown as numbers and percentages (%). Quantitative variables are presented as means and standard deviations (SD) for parametric data or as medians and interquartile range (p25–p75) for non-parametric data. The recorded value of hs-CRP was utilized in two ways during the statistical analyses; first as a continuous variable and second as a categorical variable through the creation of quintiles. These quintiles were distributed as presented in Table 2. The data are presented in quintiles following the methodology of the study by van’t Kloster et al. [23].

Boxplots are represented as follows: the lower part of the box represents the 25th percentile, the middle line represents the median, and the upper part represents the 75th percentile. The vertical line defines the minimum and maximum extremes. The chi-square test or Fisher’s exact test was used to examine comparisons between categorical variables. The distribution of continuous variables by categories is analyzed using the *t*-test, ANOVA, or Mann–Whitney U test. Correlations between two continuous variables are analyzed using the Spearman test, and results are expressed as the Spearman correlation coefficient (ρ) and its *p*-value. Logistic regression results are expressed as hazard ratios (HR) and their 95% confidence interval (lower confidence limit–upper confidence limit) along with the *p*-value. All tests were performed bilaterally, and a *p*-value less than 0.05 was considered significant. All statistical analyses were conducted using SAS 9.4 software (SAS Institute, Cary, NC, USA).

## 3. Results

### 3.1. Descriptive Characteristics of the Studied Population

We obtained a total of 174 patients. This population consisted of 79% men, with an average age of 69 years (ranging from 45 to 88 years) and a mean body mass index (BMI) of 28 ± 4.6 kg/m^2^. Regarding cardiovascular risk factors, 29% were active smokers, and 44% were former smokers. Thirty-four percent of the population had diabetes, 71% had dyslipidemia, 77% had hypertension, and 28% had a family history of early CVD (i.e., before the age of 50). Atrial fibrillation, heart failure, and alcohol consumption, all factors that could contribute to low-grade systemic inflammation, were also examined. Twelve percent of the population had atrial fibrillation (permanent or paroxysmal) and 30% had heart failure with reduced ejection fraction (left ventricular ejection fraction < 40%). Chronic alcohol consumption (defined as more than three alcohol units daily) was present in 25% of the population.

As for the pre-procedure history, 9.8% of the population had previously experienced a stroke, 48.3% had significant coronary artery disease treated with angioplasty or coronary artery bypass grafting, and 19% had peripheral vascular disease. The studied patients were on antiplatelet therapy in 85.6% of cases and statin therapy for lipid-lowering in 72.4% of cases.

Regarding biological assays, the average levels of hemoglobin, platelets, and leukocytes (including lymphocytes and neutrophils) were within normal ranges. Renal function was preserved, with an average glomerular filtration rate according to the MDRD of 65.5 ± 22.2 mL/min/1.73 m^2^. The average lipid profile was within the upper limits of normal, with a total cholesterol of 172.5 ± 38 mg/dL, HDL at 51.1 ± 15.5 mg/dL. The calculated LDL was 89.9 ± 32.6 mg/dL, significantly higher than the therapeutic targets for secondary prevention of CVD were observed in 113 patients pre-procedure, accounting for 64.9% of the population. The mean hs-CRP level was 3.4 ± 3 mg/L, and the mean fibrinogen was 3.8 ± 0.9 g/L, both within the upper limits of normal. Table 3 describes the characteristics of this population.

Demographic data and cardiovascular risk factors were similar in both groups, except for alcohol consumption. In the cancer-free group, 33% consumed alcohol daily, versus 16% in the cancer group. The cancer group had more comorbidities: atrial fibrillation was present in 15% versus 9%, and heart failure with reduced ejection fraction in 26% versus 8%. Patients who developed cancer presented with more history of coronary heart disease, 55% compared with 41% in the cancer-free population. In terms of chronic therapy, the use of statins and antiplatelet agents was higher in the cancer group at 93% and 80%, respectively, compared with 78% and 64% in the cancer-free population. Biological data showed no differences between groups.

### 3.2. Comparative Analyses

**Figure 2:**
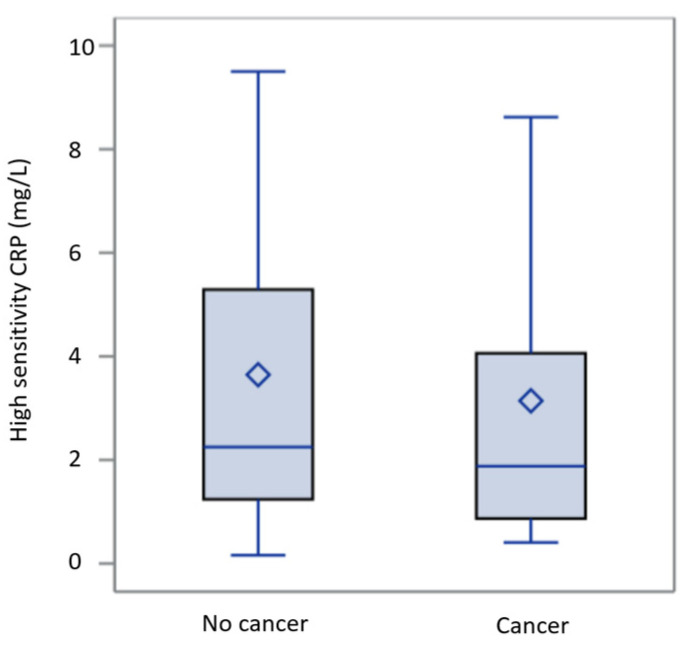
Levels of hs-CRP in cancer and non-oncologic populations.

Comparing the oncologic and non-oncologic populations revealed higher chronic alcohol consumption (*p* = 0.048) and follow-up mortality rate (*p* < 0.001) in the oncologic group. The non-oncologic group had more antiplatelet treatments (*p* = 0.08) and statin use (*p* = 0.027). No significant differences were found in the history of strokes, peripheral vascular diseases, coronary artery disease, or one-year mortality. Hs-CRP levels (*p* = 0.28), fibrinogen levels (*p* = 0.48), and leukocyte count (*p* = 0.248) were also similar between groups. There was no statistically significant difference between the levels of hs-CRP in these two populations (Figure 2) and their distribution in the various CRP quintiles described above.

There was no statistically significant difference in the development of cancers in the different CRP quintiles. However, there was a higher mortality rate at 1 year and during follow-up for the 4th and 5th quintiles, and at 1 year only (although there is a clear trend for mortality during follow-up as well) for the 3rd quintile (Table 4).

Hs-CRP levels were significantly higher in patients who died within a year (*p* = 0.01). Deaths within a year were more frequent in the highest two CRP quintiles (*p* = 0.08 and *p* = 0.004). Similar results were observed for overall follow-up mortality, with more deaths in the highest two quintiles (*p* = 0.034 and *p* = 0.045). Cancer development showed no significant difference across the CRP quintiles. However, the mortality rates at 1 year and during follow-up were higher for the 4th and 5th quintiles. The 3rd quintile only showed a statistically significant high mortality rate at 1 year, with a trend toward increased follow-up mortality. Fewer patients with heart failure were found in these last three quintiles, with significance increasing from the 3rd to the 5th. However, there was no difference regarding patients with atrial fibrillation or alcohol consumption. A strong association was observed with the fibrinogen level, but not with the leukocyte count. The results comparing the parameters across the hs-CRP quintiles are shown in Table 4.

### 3.3. Distribution and Correlation of hs-CRP Values

Hs-CRP levels, analyzed as a continuous variable, were higher in active or former smokers (*p* = 0.018) and those with dyslipidemia (*p* = 0.049). No statistically significant differences were found in hypertensive, diabetic individuals, those with an early family history of CVD, or chronic alcohol consumers (respectively, *p* = 0.298; *p* = 0.72; *p* = 0.75; *p* = 0.14). Patients with heart failure had lower hs-CRP levels (*p* < 0.001). No significant difference was observed in atrial fibrillation patients (*p* = 0.64). Hs-CRP levels were similar in populations with or without coronary artery disease and stroke history (*p* = 0.49 and 0.45), but higher in those with peripheral vascular disease (*p* = 0.019).

Antiplatelet or statin use did not significantly affect hs-CRP distribution (*p* = 0.7 and *p* = 0.61). A significant correlation existed between hs-CRP and fibrinogen (*p* < 0.001, R = 0.47). No significant correlations were found with age, BMI, or white blood cell counts, though the latter approached significance (*p* = 0.068).

### 3.4. hs-CRP as a Predictive Variable

**Figure 3:**
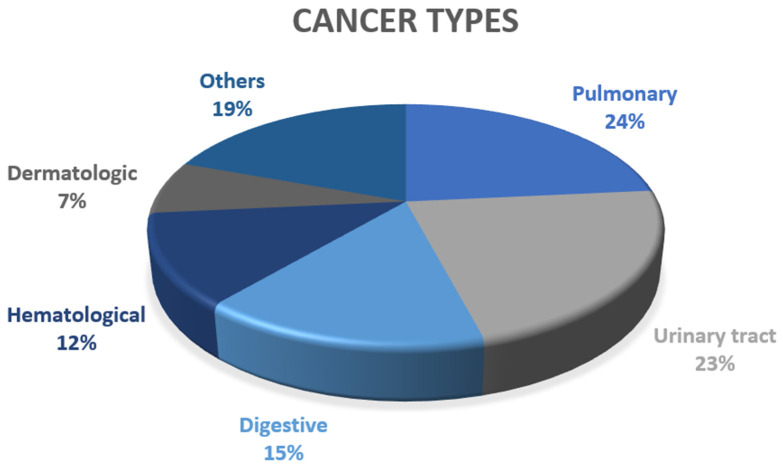
Figure representing the types of cancer developed.

Patients were followed for an average of 6.3 ± 0.7 years. In those who developed cancer, it appeared after 2.8 ± 1.9 years on average. Ninety-eight cancers were recorded, distributed as follows (Figure 3): 23 in the pulmonary system, 22 in the urological system, 15 in the digestive system, 12 in the hematological system, 7 in the dermatological system, and 19 in other locations (including gynecological, endocrinological, ears-nose-throat, neurological, and unknown).

Hs-CRP, both as an absolute and as a categorical variable, was not predictive of the development of a neoplastic condition during the follow-up of these patients. However, the levels of hs-CRP were predictive of mortality at 1 year and during follow-up (Table 5).

Antiplatelet and statin use were associated with reduced cancer risk and showed trends toward improved survival. Antiplatelet therapy significantly decreased cancer development risk by 60% (HR 0.4, *p* < 0.001) and suggested improved survival (HR 0.4, *p* = 0.07). Statin use significantly reduced cancer risk by 45% (HR 0.55, *p* = 0.009) and indicated a non-significant trend toward prolonged survival (HR 0.60, *p* = 0.096). These findings are summarized in Table 6.

## 4. Discussion

Our study revealed several key findings. Higher hs-CRP levels were not associated with increased cancer risk, but were linked to higher mortality. Fewer heart failure patients were observed in the higher hs-CRP quintiles, and a strong, expected association existed between fibrinogen and hs-CRP levels, as these are both inflammation biomarkers. Antiplatelet and statin use were associated with reduced cancer risk and suggested improved survival trends.

### 4.1. Neoplasia and hs-CRP

Our study compared two matched cohorts: patients who developed neoplasia and those who remained cancer-free. Unlike recent studies [20,23,24,25], our research did not demonstrate a significant difference in hs-CRP levels, whether analyzed as a continuous variable or in quintiles (categorical variable), based on the design of the van’t Klooster study [23]. Survival analyses, conducted through Cox regression, did not reveal a relationship between hs-CRP levels and the incidence of neoplastic conditions during the follow-up of these patients. These results differ from the van’t Klooster study, which, to our knowledge, is the only study conducted on the relationship between CRP levels and the incidence of cancer in patients with established CVD. Indeed, the latter study highlighted a relationship between CRP concentration and the risk of developing cancer, particularly lung cancer. However, it should be noted that the matching performed in our study allowed the selection of patients with similar demographic data (age and BMI) and cardiovascular risk factors (smoking, hypertension, diabetes) in both groups, making the population without neoplastic conditions quite like its oncologic counterpart.

Si et al. demonstrated that genetically determined chronic low-grade inflammation was strongly associated with various diseases in the UK Biobank and FinnGen populations. However, these associations were not consistently replicated across different populations, suggesting diverse effects of CRP [26]. While elevated hs-CRP levels have been linked to cancer and cardiovascular risk, evidence for a causal relationship between CRP and these diseases remains limited. This highlights the complex nature of CRP’s role in disease development and the need for further research to establish causality [27].

Common factors associated with chronic low-grade inflammation (obesity, diabetes, hypertension, smoking) may contribute to similar inflammation levels between groups. In our study, active or former smoking was linked to higher hs-CRP levels. Van’t Klooster et al. found no relationship between CRP and total cancer risk in never-smokers [23]. Similarly, Muller et al. reported no association between CRP levels and lung cancer in never-smokers [28]. These findings suggest that smoking-related cancer risk may involve pathophysiological pathways beyond inflammation alone.

Our study highlighted a significant association between hs-CRP levels and mortality rates, both at 1 year and during follow-up, independent of cause. This relationship was observed when analyzing CRP as both a continuous and categorical variable (top three quintiles). These findings align with current literature, such as the VISTA-16 study secondary analysis, which showed a correlation between hs-CRP levels and increased risk of major cardiovascular events as well as all-cause mortality following acute coronary syndrome [10]. Due to technical limitations, we could not differentiate between cardiovascular and other causes of death, warranting further investigation in future analyses.

### 4.2. Cardiovascular Risk Factors, Comorbidities, and hs-CRP

Atherosclerosis and CVD are known to induce low-grade systemic inflammation, leading to minor elevations in hs-CRP levels [6,7,8]. Considering our entire population had confirmed CVD, we focused on other potential factors associated with hs-CRP elevation. Among cardiovascular risk factors, only smoking status (former or current) demonstrated a significant association with CRP concentration, consistent with previous findings in the literature. In our study, patients with dyslipidemia showed higher continuous CRP levels, but this difference was not significant when comparing CRP quintiles. This finding contrasts with recent research demonstrating that inflammation is independently associated with CVD, regardless of atherogenic lipid levels [17].

### 4.3. Chronic Treatment with Antiplatelet Agents and Statins

Our study revealed a statistically significant higher proportion of patients on antiplatelet agents and statins in the non-neoplastic group. Survival analyses demonstrated a significantly lower probability of cancer development in patients receiving antiplatelet or statin treatment. This finding aligns with meta-analyses suggesting that long-term (4–6 years) low-dose aspirin use may reduce cancer risk [29,30]. Recent studies and ongoing trials are examining the reduction of atherosclerosis using anti-inflammatory drugs [31]. The CANTOS study showed a 15% reduction in cardiovascular events with an anti-IL1β antibody [32]. Other studies are targeting IL6 and IL17A or using anti-inflammatory drugs such as colchicine or methotrexate [31]. A meta-analysis suggests the potential of NSAIDs in cancer prevention and management [33]. Ongoing trials analyzing TNF antagonists for ameliorating the adverse effects of other cancer therapies will be interesting to follow. Chemokine receptor blockers are also being studied as pharmaceutical targets, as chemokines are implicated in metastasis [20]. Further trials observing a reduction of cancer incidence with these treatments would be valuable.

The association between statin use and overall cancer risk remains inconclusive in the current literature [34]. Our study demonstrated a significantly lower probability of cancer development with prior statin treatment. Although we could not directly establish this relationship, the association between statins and CRP levels has been well-documented in the literature. A meta-analysis confirmed the efficacy of statins in reducing both CRP and hs-CRP levels [35]. This meta-analysis suggests that statins have an anti-inflammatory effect, thereby reducing the risk of cancer when used. Statins probably play an important role in this complex relationship between inflammation, CVD, and cancers.

### 4.4. hs-CRP and Heart Failure

Our findings regarding heart failure patients and hs-CRP levels contradict the established literature. Current research consistently shows that elevated hs-CRP levels are associated with heart failure and worse outcomes. For example, a study showed that in patients with acute heart failure, hs-CRP levels were independently associated with an increased risk of long-term death and total heart failure admissions [36].

Contrary to these findings, our data unexpectedly revealed fewer heart failure patients in the higher hs-CRP quintiles. This discrepancy warrants careful consideration of potential confounding factors or methodological differences. Possible explanations include variations in patient demographics, comorbidities, medication use, or timing of hs-CRP measurements.

## 5. Limitations

One of the main limitations is the single measurement of hs-CRP in our patients. Current recommendations state that two measurements should be taken 2 weeks apart to reduce intra-individual variability, exclude intercurrent inflammatory processes, and increase measurement stability [2]. As our study was retrospective, these additional measurements could not be collected.

Second, the size of our population was small. We did not have sufficient numbers of histological cancer subtypes to allow for relevant statistical analyses within these subgroups.

Third, although our follow-up duration was comparable to other studies on this subject, it might have been insufficient to detect occult cancers that were not documented by the end of patient follow-up. Longer patient follow-up would yield more rigorous results.

Finally, additional data, such as the duration of antiplatelet or statin treatment before the coronary angiography procedure, would have been valuable. Unfortunately, this information was not feasible to obtain in this retrospective study, as it was not documented in medical records, and most patients were unable to provide this information.

## 6. Perspectives

Larger-scale studies are needed to better understand the complex relationships between low-grade systemic inflammation, CVD, and cancer. Future research should investigate causal links between inflammation biomarkers, CVD, and cancer development. Exploring potential new therapeutic targets and examining the effects of antiplatelet and statin treatments could provide valuable insights. These studies may lead to improved prevention and treatment strategies.

## 7. Conclusions

Our study of 174 patients with CVD provides insights into the relationships between hs-CRP, cancer development, and mortality. Hs-CRP levels did not predict cancer occurrence, but were significantly associated with 1-year and long-term all-cause mortality. Our results revealed significant associations between smoking and dyslipidemia with elevated hs-CRP levels, highlighting the complex interplay between cardiovascular risk factors and systemic inflammation. We observed a higher prevalence of antiplatelet and statin use in the cancer-free group, associated with a lower probability of developing cancer. This unexpected finding suggests potential protective effects of these medications against cancer development in CVD patients.

These observations contribute to our understanding of inflammation, cardiovascular disease, and cancer. While hs-CRP did not prove to be a cancer predictor, it reinforces its importance as a mortality risk marker. Our findings open new avenues for research into the potential cancer-protective effects of cardiovascular medications. Future large-scale prospective studies are needed to elucidate these relationships and explore underlying mechanisms, potentially leading to improved risk stratification and management strategies for cardiovascular disease patients.

## Figures and Tables

**Table 1:** Matching criteria. BMI, Body mass index.

Matching Criteria	Categories
Age	40–59, 60–79, 80–99
Sex	Male, Female
BMI	<18.5, 18.5–30, >30
Smoking Status	Smoker, Former Smoker, Non-Smoker
Diabetes	Yes, No
Hypertension	Yes, No

**Table 2:** Quintile distribution according to the level of hs-CRP.

**Quintile**	**High-Sensitivity CRP (mg/L)**
Quintile 1	0.16–0.811
Quintile 2	0.812–1.58
Quintile 3	1.59–3.19
Quintile 4	3.2–6.46
Quintile 5	6.47–9.5

**Table 3:** Descriptive characteristics of the studied population.

Demographic Data	Overall Population (N = 174)	Patients Who Did Not Develop Cancer (N = 87)	Patients Who Developed Cancer (N = 87)
Age, years	68.83 ± 8.87	67.79 ± 9.13	69.86 ± 8.52
Male gender, *n* (%)	138 (79.31)	69 (79.31)	69 (79.31)
BMI, kg/m^2^	28.26 ± 4.6	28.27 ± 4.99	28.25 ± 4.2
**Cardiovascular Risk Factors**			
Active smoking, *n* (%)	51 (29.31)	26 (29.89)	25 (28.74)
Former smoking, *n* (%)	76 (43.68)	38 (43.68)	38 (43.68)
Diabetes, *n* (%)	60 (34.48)	30 (34.48)	30 (34.48)
Dyslipidemia, *n* (%)	124 (71.26)	61 (70.11)	63 (72.41)
Hypertension, *n* (%)	134 (77.01)	68 (78.16)	66 (75.86)
Family history of early CVD, *n* (%)	48 (27.59)	25 (28.74)	23 (26.44)
Alcohol consumption, *n* (%)	43 (24.71)	14 (16.09)	29 (33.33)
**Comorbidities**			
Atrial fibrillation, *n* (%)	21 (12.07)	13 (14.94)	8 (9.2)
Heart failure with reduced EF, *n* (%)	30 (17.24)	23 (26.44)	7 (8.05)
**Medical History**			
Stroke, *n* (%)	17 (9.77)	8 (9.2)	9 (10.34)
Coronary artery disease, *n* (%)	84 (48.28)	48 (55.17)	36 (41.38)
Peripheral vascular disease, *n* (%)	33 (18.97)	19 (21.84)	14 (16.09)
**Chronic Therapy**			
Antiplatelet agent, *n* (%)	149 (85.63)	81 (93.1)	68 (78.16)
Statin, *n* (%)	126 (72.41)	70 (80.46)	56 (64.37)
**Biological Data**			
Urea, mg/dL	45.06 ± 19.18	44.74 ± 20.96	45.38 ± 17.32
Creatinine, mg/dL	1.22 ± 0.79	1.23 ± 0.99	1.22 ± 0.54
GFR (MDRD), ml/min/1.73 m^2^	65.51 ± 22.18	67.55 ± 22.83	63.44 ± 21.44
Hemoglobin, g/dL	14.42 ± 1.6	14.51 ± 1.49	14.33 ± 1.72
Platelets, 1000/µL	228.45 ± 59.45	221.43 ± 53.74	235.55 ± 64.26
Leukocytes, 1000/µL	8.06 ± 2.2	7.87 ± 2.03	8.26 ± 2.36
Lymphocytes, 1000/µL	1.97 ± 0.77	1.91 ± 0.8	2.05 ± 0.73
Neutrophils, 1000/µL	5.15 ± 1.91	5.09 ± 1.8	5.2 ± 2.02
High-sensitivity CRP, mg/L	3.39 ± 3.05	3.65 ± 3.17	3.14 ± 2.92
Fibrinogen, g/L	3.76 ± 0.92	3.71 ± 0.91	3.81 ± 0.92
Total cholesterol mg/dL	172.45 ± 38.04	174.24 ± 36.97	170.65 ± 39.21
HDL cholesterol, mg/dL	51.14 ± 15.54	50.28 ± 14.11	52 ± 16.91
LDL cholesterol (calculated), mg/dL	89.88 ± 32.58	91.48 ± 32.67	88.26 ± 32.59

BMI, Body mass index; CVD, Cardiovascular disease; EF, Ejection fraction; GFR, Glomerular filtration rate; CRP, C-reactive protein; HDL, High density lipoprotein; LDL, Low density lipoprotein.

**Table 4:** *p*-values for differences in clinical parameters by hs-CRP quintiles.

	Q3	Q4	Q5
Cancer development	*p* = 0.159	*p* = 0.220	*p* = 0.133
Mortality at 1 year	*p* = 0.021	*p* = 0.008	*p* = 0.004
Mortality during follow-up	*p* = 0.061	*p* = 0.034	*p* = 0.045
Heart failure	*p* = 0.003	*p* = 0.002	*p* = 0.000
Atrial fibrillation	*p* = 0.446	*p* = 0.593	*p* = 0.608
Chronic alcohol consumption	*p* = 0.301	*p* = 0.172	*p* = 0.109
Fibrinogen	*p* = 0.000	*p* = 0.000	*p* = 0.000
Total leucocytes	*p* = 0.221	*p* = 0.087	*p* = 0.109

Q3–Q5 represent hs-CRP quintiles from middle to highest. The *p*-values in each cell indicate statistical significance of differences for the respective parameter in each hs-CRP quintile compared to the lower quintiles.

**Table 5:** Hazard ratios, 95% confidence intervals, and *p*-values for mortality in patients with higher and lower values of hs-CRP.

	HR	LCL	UCL	*p*-Value
1 year mortality	1.57	1.11	2.22	0.011
Follow-up mortality	1.11	1.02	1.21	0.021

HR, hazard ratio; LCL, lower confidence limit; UCL, upper confidence limit.

**Table 6:** Hazard ratios, 95% confidence intervals, and *p*-values for cancer development and prolonged survival in patients with antiplatelet or statin therapy.

	HR	LCL	UCL	*p*-Value
**Antiplatelet**				
Cancer development	0.40	0.24	0.67	<0.001
Prolonged survival	0.40	0.21	0.78	0.07
**Statin**				
Cancer development	0.55	0.36	0.86	0.009
Prolonged survival	0.60	0.39	1.09	0.096

HR, hazard ratio; LCL, lower confidence limit; UCL, upper confidence limit.

## Data Availability

The data presented in this study are available on request from the corresponding author.

## Abbreviations

CVD	Cardiovascular disease
LDL	Low density lipoprotein
CRP	C-reactive protein
Hs-CRP	High-sensitivity CRP
AHA	American Heart Association
BMI	Body mass index
HR	Hazard ratio
